# Burden of maternal and fetal outcomes among pregnant cancer survivors during delivery hospitalizations in the United States

**DOI:** 10.1038/s41598-022-13852-4

**Published:** 2022-06-15

**Authors:** Muni Rubens, Venkataraghavan Ramamoorthy, Anshul Saxena, Peter McGranaghan, Sandeep Appunni, Md Ashfaq Ahmed, Zhenwei Zhang, Shelbie Burchfield, Raees Tonse, Emir Veledar, Rupesh Kotecha

**Affiliations:** 1 Miami Cancer Institute, Baptist Health South Florida, Miami, FL USA; 2 Baptist Health South Florida, Miami, FL USA; 3 Government Medical College, Calicut, Kerala India; 4 Florida International University, Miami, FL USA; 5 Department of Radiation Oncology, Miami Cancer Institute, Miami, FL USA

**Keywords:** Cancer, Oncology

## Abstract

Existing studies on pregnancy-related outcomes among cancer survivors are limited by sample size or specificity of the cancer type. This study estimated the burden of adverse maternal and fetal outcomes among pregnant cancer survivors using a national database. This study was a retrospective analysis of National Inpatient Sample collected during 2010–2014. Multivariate regression models were used to calculate odds ratios for maternal and fetal outcomes. The study included a weighted sample of 64,506 pregnant cancer survivors and 18,687,217 pregnant women without cancer. Pregnant cancer survivors had significantly higher odds for death during delivery hospitalization, compared to pregnant women without cancer (58 versus 5 deaths per 100,000 pregnancies). They also had higher odds of severe maternal morbidity (aOR 2.00 [95% CI 1.66–2.41]), cesarean section (aOR 1.27 [95% CI 1.19–1.37]), labor induction (aOR 1.17 [95% CI 1.07–1.29]), pre-eclampsia (aOR 1.18 [95% CI 1.02–1.36]), preterm labor (aOR 1.55 [95% CI 1.36–1.76]), chorioamnionitis (aOR 1.45 [95% CI 1.15–1.82]), postpartum infection (aOR 1.68 [95% CI 1.21–2.33]), venous thromboembolism (aOR 3.62 [95% CI 2.69–4.88]), and decreased fetal movements (aOR 1.67 [95% CI 1.13–2.46]). This study showed that pregnancy among cancer survivors constitutes a high-risk condition requiring advanced care and collective efforts from multiple subspecialties.

## Introduction

Advances in cancer treatment have significantly increased survival rates among women in the reproductive age group. Complications associated with cancer and its treatment can have significant adverse effects on maternal and fetal outcomes during subsequent pregnancies^1^. A study using the Scottish Cancer Registry showed that obstetrical complications such as post-partum hemorrhage, preterm delivery, cesarean section and assisted delivery were significantly higher among women with cancer compared to non-cancer patients^2^. Similarly, another study using the North Carolina Central Cancer Registry showed that the risk for preterm birth, low birth weight, and cesarean delivery were significantly higher among women with cancer^3^. In addition to these adverse associations, the symptoms of cancer often resemble normal physiological changes in pregnancy, leading to difficulty in diagnosing cancer during pregnancy^4–6^. The effects of these changes increase both maternal and fetal adverse outcomes among pregnant cancer survivors^7^. The fetus is as vulnerable as the pregnant woman to the adverse effects of cancer and antineoplastic treatment. In spite of scientific advancements and newer cancer treatments with minimal adverse effects to the growing fetus^8,9^, adverse outcomes such as growth retardation and preterm deliveries are still fairly common^10^. Given these adverse maternal and fetal outcomes, there is a clear need for studies to estimate the national burden of these problems to better define the scope of the issue.

Most of the previously published studies are either small scale, with limited sample sizes, or limited to specific types of cancers^11–16^, and large, nationally representative studies are scarce. Hence, the objective of this study was to determine nationally representative estimates of maternal and fetal outcomes among cancer survivors in the US, using the National Inpatient Sample (NIS), the largest database of hospitalization records in the country.

## Methods

This study was a cross-sectional analysis of National Inpatient Sample (NIS) data collected during the years 2010–2014. NIS was developed by the Agency for Healthcare Research and Quality (AHRQ) as a part of the Healthcare Cost and Utilization Project (HCUP). NIS is the largest all payer inpatient database and includes stratified sample of discharge data from 20% of all community hospitals within the US. Stratification is based on hospital location, bed size, hospital region, teaching status, and ownership. The NIS includes 20% of stratified sample of discharges from all US community hospitals located in the states participating in HCUP and covers more than 97% of the US population. HCUP redesigned the NIS data in 2012 to improve national estimates. It was changed to sample discharge records from all HCUP-participating hospitals, rather than a sample of hospitals from which all discharges were retained. Annually, more than 35 million weighted hospitalizations are captured by the NIS. Any hospital was considered a teaching hospital when it had a residency program approved by the Accreditation Council for Graduate Medical Education, was included in the Council of Teaching Hospitals, or had a resident to bed ratio of 0.25 or higher. Appropriate sample weights were used to obtain national estimates. The weights were calculated based on stratifying the hospitals by the following variables: census division, urban/rural location, teaching status, bed size, and ownership. The weights were estimated for each stratum, by calculating the ratio of overall discharges to the number of NIS discharges in those stratums. International Classification of Diseases, Ninth Revision, Clinical Modification (ICD-9-CM) diagnosis and procedure codes were used for reporting diagnoses and procedures during hospitalizations. Using previously validated methods^17^, we used ICD-9-CM codes for identifying women who underwent delivery during hospitalizations irrespective of their age. We used the ICD-9-CM diagnosis and procedure codes and diagnosis-related group (DRG) codes described in Supplementary Table 1 for identifying delivery hospitalizations. Within this sample, we identified cancer survivors using Clinical Classifications Software (CCS) codes 11-43, which indicate malignancies^18^. However, we could not identify whether patients were having concurrent malignancies or cancer treatments while being pregnant, or whether they were childhood cancer survivors. We followed the Strengthening the Reporting of Observational Studies in Epidemiology (STROBE) guidelines for reporting our findings.

### Patient and hospital characteristics

A number of demographic variables such as age, race, insurance coverage, and income were extracted from the database. ICD-9-CM diagnosis and procedure codes were used for identifying Elixhauser comorbidity index^19,20^, multiple pregnancies, previous cesarean section, pre-existing diabetes, hypertension, chronic renal disease, alcohol and substance use, depression, and psychiatric disorders (Supplementary Table 1). Hospital characteristics such as hospital location, region, bed size, and teaching status were also extracted from this database.

### Maternal and fetal outcomes

We used ICD-9 codes to identify adverse maternal and fetal outcomes (Supplementary Table 2). We estimated overall maternal morbidity using the maternal morbidity composite outcome developed by the Centers for Disease Control and Prevention^21,22^. We also identified adverse maternal outcomes, such as maternal death, cesarean delivery, induction of labor, length of stay, pregnancy-related hypertension, eclampsia, antepartum and postpartum hemorrhage, gestational diabetes, preterm labor, premature rupture of membrane, and chorioamnionitis, and fetal outcomes such as poor fetal growth, excessive fetal growth, fetal distress, fetal abnormalities, decreased fetal movements, and stillbirth. All mothers with multiple births were considered as single delivery hospitalizations. In this study, maternal mortality was defined as death during delivery hospitalizations due to any cause. The study was exempt from institutional review board approval as it uses previously collected deidentified data stored in NIS.

### Statistical analyses

Statistical analyses were performed using SAS version 9.4 (SAS Institute Inc., Cary, NC) using procedures that accounted for complex sampling design and clustering of the NIS^23^. To account for the redesigning of NIS data in 2012, we used trend weight (TRENDWT) for the years 2010 to 2011 and regular discharge weight (DISCWT) for the years 2012 to 2014. Descriptive statistics were calculated to understand demographics, hospital characteristics, and maternal and fetal outcomes, and were reported in terms of mean, percentages and standard errors. Rao-Scott χ^2^ tests were used for categorical variables, while Mann–Whitney U tests were used for continuous variables. Multivariate regression models were used to calculate odds ratios (ORs) and 95% confidence intervals (CIs) for all binary maternal and fetal outcomes. Regression models also included maternal age, race, median household income, Elixhauser comorbidity index, hospital region, hospital location (urban or rural), hospital teaching status and year. Variables which were significant at *P* < 0.01 in the bivariate analysis were included in the final model. Percent of missing data was small, and since data were not missing completely at random, we used the NOMCAR option during the regression analysis. All reported estimates are weighted estimates. Statistical significance was set at *P* < 0.05 and all tests were 2 sided.

### Ethics approval and consent to participate

The study was reviewed by the Miami Cancer Institute’s Institutional Review Board, which exempted the study from institutional review board approval and waived the requirement for informed consent because it uses previously collected deidentified data stored in NIS. We followed the Strengthening the Reporting of Observational Studies in Epidemiology guidelines for reporting our findings.

## Results

The NIS sample included for this study had 3,814,715 weighted delivery-related hospitalizations, of which 13,109 women were cancer survivors, while 3,801,606 did not have a concomitant or previous cancer diagnosis. Using appropriate sample weights for determining national estimates for the entire US population, we found that there were 64,506 pregnant cancer survivors and 18,687,217 pregnant women without cancer. We reported only weighted results because they were more meaningful and there were no significant changes due to inclusion of sample weights.

### Patient characteristics

The mean age of delivery was 30.5 years for women who were cancer survivors and 28.0 years for women without cancer (Table 1). Pregnant cancer survivors were more likely to be white patients (63.5% versus 52.9%) and less likely to be black (12.3% versus 14.5%), Hispanic (16.2% versus 21.6%), Asian or Pacific Islander (4.1% versus 5.4%), or Native American patients (0.45% versus 0.80%). Pregnant cancer survivors were more likely to be Medicare (1.8% versus 0.7%) and private insurance beneficiaries (57.5% versus 49.8%) and less likely to be Medicaid beneficiaries (35.9% versus 43.9%). Pregnant cancer survivors were more likely to have multiple births (2.8% versus 1.8%), previous cesarean Sects. (18.9% versus 17.2%), pre-existing diabetes (1.6% versus 1.0%), chronic renal disease (0.60% versus 0.28%), pre-existing hypertension (4.0% versus 2.3%), depression (4.8% versus 2.3%), alcohol or substance abuse (3.2% versus 1.8%), psychiatric disorders (6.1% versus 2.5%), and higher Elixhauser comorbidity index (3.1% versus 0.70%). There were no significant differences in smoking or obesity between the two groups.

**Table 1:** Baseline characteristics of pregnant cancers survivors and pregnant women without cancers, n = 18,751,723.

Characteristics	Cancer survivors	Without cancer	*P* value
Unweighted sample	13,109	3,801,606	
Weighted sample	64,506	18,687,217	
Age in years, mean (SE)	30.5 (0.08)	28.0 (0.03)	< 0.001
**Race, % (SE)**			< 0.001
White	63.5% (0.97)	52.9% (0.52)	
Black	12.3% (0.53)	14.5% (0.30)	
Hispanic	16.2% (0.83)	21.6% (0.47)	
Asian or Pacific Islander	4.1% (0.27)	5.4% (0.20)	
Native American	0.45% (0.07)	0.80% (0.05)	
Other	3.5% (0.22)	4.8% (0.15)	
**Insurance type, % (SE)**			< 0.001
Medicare	1.8% (0.12)	0.7% (0.04)	
Medicaid	35.9% (0.91)	43.9% (0.45)	
Private insurance	57.5% (0.95)	49.8% (0.48)	
Self-pay	1.8% (0.16)	2.6% (0.10)	
No charge^a^	0.13% (0.03)	0.12% (0.03)	
Other^b^	2.9% (0.17)	2.9% (0.08)	
**Median household income for patient’s zip code, % (SE)**			< 0.001
Quartile 1	24.2% (0.73)	27.7% (0.47)	
Quartile 2	22.7% (0.48)	25.0% (0.32)	
Quartile 3	25.4% (0.50)	25.2% (0.30)	
Quartile 4	27.7% (0.79)	22.1% (0.57)	
Elixhauser comorbidity index ≥ 4, % (SE)	3.1% (0.16)	0.70% (0.01)	< 0.001
Multiple birth, % (SE)	2.8% (0.16)	1.8% (0.02)	< 0.001
Previous cesarean delivery, % (SE)	18.9% (0.36)	17.2% (0.05)	< 0.001
Preexisting diabetes mellitus, % (SE)	1.6% (0.11)	1.0% (0.01)	< 0.001
Chronic renal disease, % (SE)	0.60% (0.07)	0.28% (0.01)	< 0.001
Preexisting hypertension, % (SE)	4.0% (0.18)	2.3% (0.02)	< 0.001
Depression, % (SE)	4.8% (0.20)	2.3% (0.04)	< 0.001
Alcohol or substance abuse, % (SE)	3.2% (0.20)	1.8% (0.03)	< 0.001
Psychiatric disorders^c^, % (SE)	6.1% (0.23)	2.5% (0.03)	< 0.001
Smoking, % (SE)	1.1% (0.18)	1.2% (0.03)	0.622
Obesity, % (SE)	8.1% (0.22)	9.6% (0.01)	0.162

^a^Care provided as charity, courtesy, or free of charge.

^b^This category includes Worker’s Compensation, the Civilian Health and Medical Program of the Uniformed Services, the Civilian Health and Medical Program of the Department of Veterans Affairs, Title V, and other government programs.

^c^Includes anxiety, adjustment, eating, mood, personality, and psychotic disorders.

### Hospital characteristics

There were differences in hospital characteristics such as region, location, bed size, and teaching status between pregnant cancer survivors and those without cancer (Table 2). Pregnant cancer survivors were more likely to be admitted to hospitals in Northeast region (20.1%), urban locations (92.2%), teaching (58.8%), and large (63.5%) hospitals.

**Table 2:** Characteristics of Hospitals where Pregnant Cancers Survivors and Pregnant Women without Cancers were Hospitalized for Delivery, n = 18,751,723.

Characteristics	Cancer Survivors	Without Cancer	*P* value
**Region, % (SE)**			< 0.001
Northeast	20.1% (0.98)	16% (0.41)	
Midwest	21.2% (0.83)	21.2% (0.47)	
South	35.9% (1.18)	38.1% (0.7)	
West	22.8% (0.86)	24.7% (0.63)	
**Location, % (SE)**			< 0.001
Rural	7.8% (0.68)	11.4% (0.48)	
Urban	92.2% (0.68)	88.6% (0.48)	
**Bed size**^**a**^**, % (SE)**			< 0.001
Small	11.7% (0.59)	12.4% (0.32)	
Medium	24.8% (0.91)	28.2% (0.58)	
Large	63.5% (1.04)	59.4% (0.64)	
**Teaching status, % (SE)**			< 0.001
Teaching	58.8% (2.2)	47.1% (1.39)	
Non-teaching	41.2% (2.2)	52.9% (1.39)	

^a^Bed size categories are based on hospital beds and are specific to the hospital's location and teaching status. For details of categorization, please see: https://www.hcup-us.ahrq.gov/db/vars/hosp_bedsize/nisnote.jsp.

### Cancer types

The most common cancers recorded in pregnant cancer survivors were lymphomas and leukemias (31.28%), female genital organ cancers (27.2%), urinary cancers (15.52%), and breast cancer (9.17%; Table 3).

**Table 3:** Cancer types and frequencies among pregnant cancer survivors, n = 64,506.

Cancer types	% (SE)
Stomach	0.18% (0.04)
Colon	0.93% (0.08)
Liver	0.24% (0.04)
GI organs and peritoneum	0.99% (0.12)
Lung and bronchus	0.5% (0.06)
Breast	9.2% (0.28)
Uterus	0.42% (0.05)
Ovary	2.4% (0.14)
Other female genital organs	27.2% (0.98)
Melanomas of skin	1.8% (0.12)
Urinary	15.5% (0.39)
Lymphomas and leukemia	31.3% (0.62)
Cancer with unspecified primary	8.4% (0.43)
Secondary malignancies	0.94% (0.08)
Malignant neoplasm without specification of site	0.09% (0.03)

### Maternal mortality

There were 58 maternal deaths per 100,000 pregnancies among cancer survivors, compared to 5 per 100,000 pregnancies among those without cancer. The odds of maternal mortality were almost 7 times higher among cancer survivors compared to those without cancer, after controlling for maternal age, race, median household income for patient's ZIP Code, insurance type, comorbidity index, hospital region, hospital location, hospital teaching status, and survey years (adjusted OR [aOR], 6.90 [95% CI 2.07–23.0]; Table 4).

**Table 4:** Obstetrical outcomes among pregnant cancers survivors and pregnant women without cancers, n = 18,751,723.

Outcome	Women, No. (SE)		*P* value
	Cancer survivors	Without cancer	
**Maternal outcome**			
Maternal death, % (SE)	0.06% (0.02)	0.005% (0.004)	0.010
Severe maternal morbidity, % (SE)	3.5% (0.17)	1.7% (0.02)	< 0.001
Cesarean delivery, % (SE)	38.5% (0.49)	33.1% (0.12)	< 0.001
Induction of labor, % (SE)	22.3% (0.41)	19.2% (0.15)	< 0.001
Length of stay, mean (SE)			
Cesarean delivery	4.3 (0.09)	3.5 (0.01)	< 0.001
Vaginal delivery	2.4 (0.03)	2.2 (0.01)	< 0.001
Length of stay > 6 days, % (SE)			
Cesarean delivery	6.9% (0.38)	3.14% (0.05)	
Vaginal delivery	1.3% (0.13)	0.66% (0.02)	
Retained placenta, % (SE)	0.63% (0.07)	0.50% (0.01)	
Gestational hypertension, % (SE)	4.3% (0.18)	4.2% (0.03)	< 0.001
Pre-eclampsia, % (SE)	5.8% (0.21)	4.4% (0.03)	
Eclampsia, % (SE)	0.84% (0.03)	0.06% (0.001)	0.525
Antepartum hemorrhage, % (SE)	1.9% (0.12)	1.5% (0.01)	0.006
Postpartum hemorrhage, % (SE)	4.3% (0.2)	3.0% (0.04)	< 0.001
Gestational diabetes, % (SE)	8.1% (0.24)	6.5% (0.05)	< 0.001
Preterm labor, % (SE)	9.1% (0.29)	6.6% (0.05)	< 0.001
Premature rupture of membranes, % (SE)	5.5% (0.23)	4.3% (0.06)	< 0.001
Chorioamnionitis, % (SE)	2.4% (0.15)	1.9% (0.04)	< 0.001
Postpartum infection, % (SE)	0.84% (0.08)	0.67% (0.02)	
Venous thromboembolism, % (SE)	1.0% (0.09)	0.21% (0.003)	< 0.001
**Fetal outcome**			
Poor fetal growth, % (SE)	3.2% (0.16)	2.6% (0.03)	< 0.001
Excessive fetal growth, % (SE)	3.1% (0.16)	2.6% (0.03)	0.002
Fetal distress, % (SE)	16.7% (0.38)	14.6% (0.14)	< 0.001
Fetal central nervous system malformations, % (SE)	0.09% (0.02)	0.06% (0.002)	0.212
Fetal chromosomal abnormalities, % (SE)	0.16% (0.03)	0.09% (0.002)	< 0.001
Suspected fetal damage due to drugs or radiation, % (SE)	0.03% (0.01)	0.03% (0.001)	0.903
Decreased fetal movements, % (SE)	1.2% (0.11)	0.72% (0.01)	< 0.001
Stillbirth, % (SE)	0.47% (0.06)	0.62% (0.01)	0.120

### Obstetrical outcomes

Pregnant cancer survivors had significantly higher odds for adverse maternal morbidity outcomes as estimated by the severe maternal morbidity indicator (aOR 2.00 [95% CI 1.66–2.41]), when compared to those without cancer (Table 5). Pregnant cancer survivors also had significantly higher odds for cesarean sections (aOR 1.27 [95% CI 1.19–1.37]) and labor inductions (aOR 1.17 [95% CI 1.07–1.29]). Hospital length of stay > 6 days was significantly higher among pregnant cancer survivors for both cesarean sections (aOR 1.93 [95% CI 1.54–2.40]) and vaginal deliveries (aOR 2.17 [95% CI 1.56–3.04]). Mean hospital length of stay was also higher among pregnant cancer survivors. The mean hospital length of stay for cesarean section and vaginal delivery were 4.3 and 2.4 days, respectively, among pregnant cancer survivors compared to 3.5 and 2.2 days, respectively, among those without cancer. Pregnant cancer survivors had significantly higher odds for pre-eclampsia (aOR 1.18 [95% CI 1.02–1.36]), preterm labor (aOR 1.55 [95% CI 1.36–1.76]), chorioamnionitis (aOR 1.45 [95% CI 1.15–1.82]), postpartum infection (aOR 1.68 [95% CI 1.21–2.33]), and venous thromboembolism (aOR 3.62 [95% CI 2.69–4.88]).

**Table 5:** Predictors of obstetrical outcomes among pregnant cancers survivors and pregnant women without cancers, n = 18,751,723.

Outcome	Crude OR (95% CI)	Adjusted OR (95% CI)^a^
**Maternal outcome**		
Maternal death	**11.05 (5.46–22.34)**	**6.90 (2.07–22.98)**
Severe maternal morbidity	**2.06 (1.87–2.27)**	**2.00 (1.66–2.41)**
Cesarean delivery	**1.26 (1.22–1.32)**	**1.27 (1.19–1.37)**
Labor induction	**1.21 (1.16–1.27)**	**1.17 (1.07–1.29)**
Length of stay > 6 days		
Cesarean delivery	**2.29 (2.05–2.56)**	**1.93 (1.54–2.40)**
Vaginal delivery	**1.99 (1.63–2.42)**	**2.17 (1.56–3.04)**
Retained placenta		1.06 (0.71–1.58)
Gestational hypertension		0.91 (0.77–1.09)
Pre-eclampsia		**1.18 (1.02–1.36)**
Eclampsia	1.23 (0.69–2.24)	0.92 (0.23–3.74)
Antepartum hemorrhage	**1.22 (1.08–1.39)**	1.04 (0.81–1.35)
Postpartum hemorrhage	**1.44 (1.31–1.57)**	1.16 (0.91–1.42)
Gestational diabetes	**1.27 (1.20–1.36)**	0.99 (0.87–1.14)
Preterm labor	**1.41 (1.32–1.50)**	**1.55 (1.36–1.76)**
Premature rupture of membranes	**1.29 (1.19–1.40)**	1.06 (0.92–1.23)
Chorioamnionitis	**1.27 (1.28–1.43)**	**1.45 (1.15–1.82)**
Postpartum infection		**1.68 (1.21–2.33)**
Venous thromboembolism	**4.89 (4.12–5.79)**	**3.62 (2.69–4.88)**
**Fetal outcome**		
Poor fetal growth	**1.27 (1.15–1.40)**	1.20 (0.97–1.48)
Excessive fetal growth	**1.20 (1.08–1.32)**	1.09 (0.89–1.33)
Fetal distress	**1.17 (1.12–1.23)**	1.07 (0.97–1.18)
Fetal central nervous system malformations	1.56 (0.89–2.72)	1.32 (0.44–3.97)
Fetal chromosomal abnormalities	**1.83 (1.18–2.83)**	0.83 (0.26–2.62)
Suspected fetal damage due to drugs or radiation	**1.07 (1.40–2.82)**	0.91 (0.13–6.32)
Decreased fetal movements	**1.72 (1.45–2.05)**	**1.67 (1.13–2.46)**
Stillbirth	0.76 (0.58–1.00)	0.90 (0.56–1.44)

^a^Adjusted for maternal age, race, quartile of median household income for patient’s zip code, Elixhauser comorbidity index, hospital region, hospital location, hospital teaching status and year.

Significant values are in bold.

Pregnant cancer survivors also had significantly higher odds for developing decreased fetal movements (adjusted OR, 1.67 [95% CI 1.13–2.46]). However, no association was found between maternal cancer and other fetal outcomes (Table 5).

## Discussion

This study showed that the odds for maternal and fetal adverse outcomes were significantly higher among pregnant cancer survivors, compared to pregnant women without cancer, or a history of cancer. The most remarkable finding included a 7-times higher odds for maternal mortality among cancer survivors. These women also had higher levels of maternal morbidity, and complications such as pre-eclampsia, preterm labor, chorioamnionitis, postpartum infections, and venous thromboembolism compared to those without cancer. Pregnant cancer survivors also had longer hospital length of stay and were more likely to undergo cesarean section and labor inductions. Pregnant cancer survivors also had greater odds for decreased fetal movements compared to those without cancer, though stillbirth was not affected. In accordance with the higher odds for maternal and fetal adverse outcomes observed in our study, we also found that pregnant cancer survivors were more likely to be admitted to larger, urban, and teaching hospitals with better treatment options. Similar to our findings, Terry et al. found that pregnant women with CNS neoplasms were more likely to be admitted to larger teaching hospitals^24^.

Although pregnant cancer survivors had a 7-times higher odds for maternal mortality (59 versus 5 deaths per 100,000 pregnancies), this may underestimate maternal mortality because only deaths that occurred during delivery hospitalizations were included, and data on peripartum and postpartum deaths were not available. However, despite the several fold increase in maternal mortality found in our study, it should be noted that absolute maternal mortality during delivery is still very uncommon among cancer survivors. The risk difference in maternal mortality between pregnant cancer survivors and pregnant women without cancer was 0.01 (95% CI 0.006–0.014).

We could not infer the specific cause for increased mortality during delivery hospitalizations among pregnant cancer survivors. Putatively, the increased odds could be due to cancer, or higher severe maternal morbidity indicators, or due to higher rates of obstetric life-threatening complications such as chorioamnionitis and venous thromboembolism. In order to estimate the effect of these life-threatening conditions on mortality, we included these factors in equations for maternal mortality in addition to other covariates. However, our results did not change significantly, and maternal mortality remained the same. Hence, we could not ascertain which of these life-threatening conditions significantly affected maternal mortality. Although we could not define the specific causes underlying the higher rates of maternal mortality from the available data, it is certain that pregnant cancer survivors have higher odds for morbidity and mortality and need additional care^25,26^. Future studies should focus on ascertaining the specific causes of higher rates of mortality in this population for developing effective interventions.

Though the majority of the outcomes in our study were also reported in previous studies among pregnant cancer survivors, we could calculate more generalizable estimates because of the nationally representative large database used for this study. Similar to studies among pregnant women with cancers, our study also showed increased odds for pre-eclampsia, chorioamnionitis, venous thromboembolism, preterm labor, cesarean section, labor induction, and increased length of stay^12–16,27^. We found that pregnant cancer survivors had 38% higher odds for cesarean section, accounting for approximately 3700 additional cesarean sections per 100,000 deliveries, and 22% higher odds for labor induction, translating to 3100 additional labor inductions per 100,000 deliveries. This could be due to the fact that cesarean section and labor induction may be recommended for reasons such as early initiation of chemotherapy or other cancer treatments. Similarly, estimated odds for other conditions accounted for 510 additional cases of chorioamnionitis, 810 additional cases of venous thromboembolism, and 2400 additional cases of preterm labor per 100,000 deliveries among pregnant cancer survivors. However, our study did not show any increased odds for antepartum hemorrhage, postpartum hemorrhage, and premature rupture of membrane^12,13,15^. Contrary to findings in a previous study^12^, our study did not show increased odds for gestational diabetes among pregnant cancer survivors.

Our study showed that 1.2% of pregnant cancer survivors had decreased fetal movements, compared to 0.72% among pregnant women without cancers. However, we did not find an increased odds for adverse fetal outcomes, such as abnormal fetal growth, fetal distress, and fetal malformations as reported in other studies done among pregnant women with cancer^12,28^. Although cancer could be associated with adverse fetal outcomes, the absence of such associations in this cohort could be due to significant improvements in pre- and perinatal screening and appropriate management. Nevertheless, the relationship between cancer treatments and decreased fetal movements should be explored in greater details in future studies. There could be several reasons for increased maternal mortality and complications among cancer survivors in our study. Chemotherapy and radiotherapy could adversely affect reproductive organs such as uterus and ovaries and produce permanent mutations in ovum leading to adverse delivery outcomes^29^. In addition, cancers of reproductive organs could result in anatomical changes to organs like the uterus leading to spontaneous abortions and other obstetric complications^30^.

### Strengths and limitations

One of the main strengths of this study was that it was one of the few large-scale studies that evaluated maternal and fetal outcomes among pregnant women with cancer at the national level. Pregnancy among cancer survivors is rare and estimating outcomes such as maternal mortality and pregnancy related complications require large sample sizes. NIS is the largest all payer inpatient database in the US and contains data from approximately 19 million weighted delivery hospitalizations and 64,000 delivered cancer survivors. This large database was helpful in estimating such rare outcomes. Because of the complex survey design, NIS represents discharges from all community hospitals within the US, thereby providing a precise estimate of maternal and fetal outcomes among pregnant women with cancers in the US.

In spite of these strengths, our study has some limitations. We used ICD-9 codes for identifying delivery hospitalizations, associated conditions, and procedures. There could be some potential coding errors and missing codes leading to misclassification bias. NIS does not record readmission and considers it as independent new admission. In addition, the unit of measurement in NIS is hospitalization and not the patient. This could have obscured the distinction between index admission and readmission. NIS lacks many cancer-related details, such as year since diagnosis, cancer staging, treatment and whether cancer was diagnosed before or during pregnancy or during childhood, or pregnancy-related details, such as use of assisted reproductive technologies. In addition, we were unable to distinguish between cancer during pregnancy, at delivery, and pre-pregnancy. This limits our understanding about the effects of these factors on maternal and fetal outcomes during delivery hospitalizations. Cancer history could be more likely to be coded during the hospitalization in the presence of an adverse pregnancy outcome, leading to differential reporting bias. The association between maternal and neonatal outcomes could not be ascertained because NIS deidentifies data and maternal and neonatal records could not be linked together, and hence, neonatal complications due to cancer among mothers could not be tracked and estimated. In addition, women who have previously experienced adverse pregnancy or birth outcomes have greater risks in successive pregnancies. However, this could not be ascertained because successive pregnancies by the same woman could not be identified. Many conditions that were not directly associated with maternal and fetal outcomes may not have been recorded in discharge data leading to differential reporting biases and imprecisions in estimating odds ratios^31,32^. Many pre-existing chronic diseases may not be recorded because of lack of direct association with obstetric or neonatal care, leading to imprecise estimation of their prevalence. Additionally, this imprecision in estimating chronic conditions may have differentially affected data from pregnant cancer survivors compared to pregnant women without cancer because cancer survivors could have received greater levels of monitoring and care before as well as during pregnancy and during delivery hospitalizations as well. We have only included NIS data collected during 2010 to 2014 because ICD-9 codes were used until 2014 and subsequently ICD-10 codes from 2015 onwards. To avoid misclassification bias due to this change we restricted our period of analysis.

## Conclusion

Though pregnancy is rare among cancer survivors, approximately 400,000 women in the reproductive-age group have history of cancer and could become pregnant^33^. Thus, there is vital need to understand this risk and prevent adverse maternal and fetal outcomes in this population. Because of significantly higher maternal and fetal adverse outcomes among pregnant cancer survivors, it is evident that pregnancy in this group constitutes a high-risk condition and requires advanced care from many subspecialties such as oncology, obstetrics, pediatrics, and critical care. Though referrals to Level 4 hospitals have significantly improved maternal outcomes for many obstetrical complications^34^, whether similar referrals could improve outcomes in women with cancer is yet to be understood.

## Supplementary Information

Supplementary Information.

## Data Availability

The dataset used in this study is publicly available for purchase from https://www.hcup-us.ahrq.gov/nisoverview.jsp.
