# Supplementary material for: Burden of maternal and fetal outcomes among pregnant cancer survivors during delivery hospitalizations in the United States
Source: Sci Rep. 2022 Jun 15;12:9989. doi: 10.1038/s41598-022-13852-4 (PMC9200828; doi:10.1038/s41598-022-13852-4)
Supplement: Supplementary file 1 — Supplementary Information. [file 41598_2022_13852_MOESM1_ESM.doc]

Supplementary Table 1. ICD-9-CM codes for baseline characteristics

| **Variables** | **ICD-9-CM codes** |
| --- | --- |
| **Delivery hospitalizations** | |
| Outcome of delivery | V27 |
| Normal delivery | 650 |
| Diagnosis-related group (DRG)  delivery codes | 370 (complicated cesarean section)  371 (uncomplicated cesarean section)  372 (complicated vaginal delivery)  373 (uncomplicated vaginal delivery)  374 (uncomplicated vaginal delivery with sterilization  and/or dilatation & curettage)  375 (vaginal delivery with operation room procedure except sterilization and/or dilatation & curettage) |
| Selected delivery related  procedures | ICD-9-CM = 720, 721, 7221, 7229, 7231, 7239,  724, 726 (forceps)  7251, 7252, 7253, 7254 (breech extraction)  7271, 7279 (vacuum extraction)  728, 729 (other specified and unspecified delivery)  7322 (internal and combined version and extraction)  7359 (other manually assisted deliveries)  736 (episiotomy)  740, 741, 742, 744, 7499 (cesarean section) |
| Exclusions | CM 630 (hydatidiform mole)  631 (other abnormal product of conception)  633 (ectopic pregnancy)  632, 634, 635, 636, 637, 638, 639, 69.01, 69.51,  74.91, 75.0 (abortion) |
| **Baseline characteristics** | |
| Multiple Births | V27.2-V27.7, 651.xx |
| Previous cesarean delivery | 654.2x |
| Pre-existing Diabetes Mellitus | 648.0x, 250.xx |
| Chronic Renal Disease | 581.xx-583.xx, 585., 587, 646.2x |
| Pre-existing Hypertension | 642.0x-642.2x, 642.7x, 401.x, 402.xx-405.xx |
| Depression | 296.2x, 296.3x, 300.4, 311, 298.0, 309.0, 309.1 |
| Alcohol and Substance Abuse | 291.xx, 292.xx, 303.xx-305.xx, 648.3x, 655.5x, 965.0x, V65.42 |
| Psychiatric Disorders |  |
| Psychotic Disorders | 295.xx-298.x, 293.81-293.82 |
| Mood Disorders | 293.83 |
| Anxiety Disorders | 293.84, 300.xx |
| Personality Disorders | 301.xx |
| Eating Disorders | 307.1, 307.51 |
| Adjustment Disorders | 309.24, 309.28, 309.3, 309.4, 309.9 |

Supplementary Table 2. ICD-9-CM codes for adverse maternal and fetal outcomes

| **Adverse maternal and fetal outcomes** | **ICD-9-CM codes** |
| --- | --- |
| Cesarean delivery | 74.0-74.2, 74.4, 74.99, 669.7x |
| Induction of Labor | 73.1, 73.4 |
| Pregnancy-related hypertension |  |
| Gestational hypertension | 642.3x, 642.9x |
| Preeclampsia | 642.4x, 642.5x, 642.6x, 642.7x |
| Antepartum Hemorrhage | 641.1x, 641.2x, 641.3x, 641.8x, 641.9x |
| Postpartum Hemorrhage |  |
| Due to atony | 666.1x |
| Not due to atony | 666.0x, 666.2x, 666.3x |
| Severe Postpartum Hemorrhage | [666.0x-666.3x] plus either blood transfusion [99.0x] or hysterectomy [68.3x-68.9] |
| Gestational diabetes | 648.8x |
| Preterm labor | 644.2x |
| Premature Rupture of Membranes | 658.1x |
| Chorioamnionitis | 658.4x |
| Poor Fetal growth | 656.5x |
| Excessive fetal growth | 656.6x |
| Fetal distress | 656.3x, 659.7x |
| Fetal abnormalities |  |
| Central nervous system malformations | 655.0x |
| Chromosomal abnormalities | 655.1x |
| Hereditary disease in family possible affecting fetus | 655.2 |
| Decreased fetal movements | 655.7x |
| Other/unspecified abnormalities | 655.8x 655.9x |
| Stillbirth | V27.1, V27.3, V27.4, V27.6, V27.7, 656.4x |
